# Evaluation of Wear Resistance in Tungsten-Doped Diamond-like Carbon Coatings (WC/C) on Coated and Uncoated Surfaces Under Starved Oil Lubrication with R452A Refrigerant

**DOI:** 10.3390/ma17225504

**Published:** 2024-11-12

**Authors:** Kasper Górny, Monika Madej, Arkadiusz Stachowiak

**Affiliations:** 1 Institute of Machines and Motor Vehicles, Poznan University of Technology, 60-965 Poznan, Poland; arkadiusz.stachowiak@put.poznan.pl; 2 Faculty of Mechatronics and Mechanical Engineering, Kielce University of Technology, al. Tysiąclecia Państwa Polskiego 7, 25-314 Kielce, Poland; mmadej@tu.kielce.pl

**Keywords:** oil/refrigerant mixture, lubricity, starved lubrication conditions, WC/C coating

## Abstract

This article assesses the potential of using a diamond-like carbon coating doped with tungsten, a-C:H:W (WC/C), on the sliding pairs of refrigeration compressors. The ability of WC/C coating to provide low wear and a low coefficient of friction was experimentally verified in a specific refrigeration compressor operating environment (lubrication with oil diluted with refrigerant) and under extreme operating conditions (starved lubrication with a small amount of oil). Conditions of starved lubrication with a substance of reduced lubricity promote a temperature increase and high mechanical (friction) stresses on the surface of the sliding pairs. These situations can hinder the effective operation of WC/C coatings. Comparative wear tests were carried out for S235JR steel samples with and without WC/C coating. It was found that the samples with the WC/C coating had the lowest wear values and the lowest friction coefficients (approximately 0.06). A low coefficient of friction suggests that even a small amount of oil (one drop) is likely sufficient to achieve mixed lubrication conditions between the tested sliding surfaces and reduce material loss. The tested WC/C coating can protect sliding friction pairs in refrigeration compressors under extreme operating conditions caused by a lack of oil. Less friction reduces the need for energy to drive the refrigeration compressor. Additionally, the significance of this research is highlighted by the fact that the wear tests were conducted using R452A, a novel, eco-friendly refrigerant.

## 1. Introduction

In recent decades, diamond-like carbon (DLC) coatings have found widespread application in various industrial and medical fields due to their exceptional wear-resistant properties. Their key advantages include high optical transmittance, low dielectric constant, low surface energy, high stiffness, a low coefficient of friction (COF), and good biocompatibility. The structure of DLC coatings comprises sp^3^ bonds characteristic of diamond and sp^2^ bonds characteristic of graphite. The properties of these coatings are strongly dependent on the ratio of sp^3^/sp^2^ hybridized bonds and the deposition technique—physical vapor deposition (PVD) and chemical vapor deposition (CVD) [1].

DLC coatings are typically deposited using plasma-assisted chemical vapor deposition (PACVD) on an adhesive interlayer, usually chromium or silicon, between the substrate and the coating [2,3]. The adhesive layer absorbs some of the high compressive stresses in the coating and protects it from delamination. DLC coatings are very interesting due to their synergistic interaction with lubricants and good adhesion to contact surfaces. Their low coefficient of friction is attributed to their specific properties (low internal stress, chemical inertness, high thermal conductivity, and excellent adhesion to the substrate) [1,4,5].

The functional properties of DLC coatings can be improved by doping them with various elements: metals or non-metals. The key mechanism for low friction in tribological pairs with DLC coatings is the presence of sp^2^ and sp^3^ carbon–carbon bonds. Sp^3^ carbon–carbon bonds (diamond) are responsible for the coating’s high hardness and wear resistance, while sp^2^ bonds (graphite) are responsible for low friction. Graphite has excellent lubricating properties, which facilitates sliding. Depending on the conditions (load, temperature, speed), the DLC coating can dynamically change its structure, adapting to the prevailing conditions in the friction zone. DLC coatings perform well in dry and humid environments and at low and high temperatures, making them versatile for many industrial applications.

Furthermore, doping DLC coatings with tungsten results in the formation of tungsten carbide (WC) inclusions in the DLC coating matrix. WC particles provide the hardness and wear resistance required for improved tribological durability. Tungsten-doped diamond-like carbon (WC/C) coatings reduce adhesive wear (resulting from the welding of surfaces), which is essential in environments where metal elements tend to “weld” together in the contact zone. These coatings are excellent in environments lacking traditional lubricants, making them ideal for applications under dry and starved lubrication conditions [1,4,5].

In addition to DLC (WC/C) coatings, other coatings are also used in the production of machine elements. In the chemical, petrochemical, marine, and pharmaceutical industries, chromium nitride (CrN) and titanium nitride (TiN) coatings provide protection against the harmful effects of aggressive substances [6,7]. In the aviation, aerospace, and metalworking industries, coatings such as titanium aluminum nitride (TiAlN) or aluminum chromium nitride (AlCrN) ensure surface stability and resistance to extreme temperatures [8]. Molybdenum disulfide (MoS_2_) coatings, on the other hand, provide a low coefficient of friction and are successfully used in the automotive, aerospace, and defense industries [9]. By applying coatings, machine elements can maintain their performance in demanding conditions, contributing to improved system reliability and safety. DLC coatings, though extremely hard, can become brittle due to internal stresses. Doping with tungsten mitigates this issue, enhancing durability in extreme conditions. Due to tungsten’s ability to reduce brittleness, WC/C coatings exhibit superior resistance to variable loads compared to TiN coatings. Additionally, WC/C offers significantly lower friction, making it ideal for dry friction conditions where minimal wear is crucial. In contrast, TiN coatings, while hard, are less flexible, making them more susceptible to chipping under high and dynamic loads. Both DLC and MoS_2_ coatings are known for their low friction coefficients. However, MoS_2_ is a solid lubricant, and environmental factors can significantly influence its performance. WC/C coatings, on the other hand, consistently maintain low friction across a broader range of conditions. Considering these factors, tungsten-doped DLC (WC/C) coatings emerge as a promising choice for applications in the refrigeration industry.

Despite their numerous advantages, tungsten-doped DLC coatings may not always perform as expected in all environments. In high-humidity environments (e.g., air with high water vapor content), DLC coatings can exhibit increased friction. This is due to the adsorption of water molecules on the surface, resulting in the deterioration of the lubricating properties. The graphitized layers (sp^2^) on the surface of the coatings can react with water, altering their tribological properties. As a result, the coefficient of friction and wear increase. Although DLC coatings have good wear resistance over a wide temperature range, at very high temperatures (>400 °C), the coatings can undergo decohesion, leading to degradation of their structure and higher friction coefficients.

The effectiveness of WC/C coatings can be limited in environments with high humidity, extreme temperatures, aggressive chemical environments, and dynamic loads. Proper coating selection for specific operating conditions is crucial to achieve optimal performance and durability of the coatings [5,10].

One promising application area for tungsten-doped DLC (WC/C) coatings is their implementation in refrigeration compressor components. Tribosystems in refrigeration compressors are lubricated with an oil-refrigerant mixture. This is problematic because the exact amount of refrigerant in the oil-refrigerant mixture is difficult to determine in actual operation. The thermodynamic parameters of the refrigeration system determine this composition, which depends on temperature and pressure [11].

Compressor failures often occur under extreme operating conditions, which can induce mixed friction and accelerated wear of the moving components. Insufficient or starved lubrication can arise when the oil-refrigerant mixture is inadequate due to oil accumulation in heat exchangers (condenser, evaporator) or other refrigeration system components. In some cases, the lubricant’s viscosity may change in low-temperature areas, which can hinder the transport of the lubricant by the refrigerant. Problems with lubricant transport can also occur during rapid fluctuations in refrigerant velocity, preventing it from transferring the required amount of oil from the refrigeration system to the compressor.

Stricter environmental regulations are pushing for refrigerants with the lowest possible global warming potential (GWP). This is especially challenging in refrigerated transport. Previously, R404A was a popular choice due to its performance, but its high GWP (3922) has led to a search for more environmentally friendly alternatives with similar capabilities. R452A, a blend of 11% R32, 59% R125, and 30% R1234yf, is a potential replacement for R404A in transport refrigeration [12]. As a hydrofluoroolefin, R452A has a lower global warming potential (GWP) of 1945 compared to R404A’s GWP of 3922. Notably, R452A’s discharge temperature is similar to that of R404A, allowing direct substitution without system modifications [13]. For R452A, polyol ester-based lubricants (POE) are recommended [14,15,16,17,18,19,20]. It is worth noting that there are reports of using WC/C coatings with POE lubricants [21]. Previous studies on coatings for refrigeration compressor friction nodes often focused on oil-free applications or those with minimal lubrication [22,23,24,25]. Various refrigerants were used, including R134a [23,24,26], R410A [24,25], R600a [23,24], R744 [25], R32 [27] or R1234yf [28,29], When oil was used, POE [27] and polyalkylene glycol-based (PAG) [26,28,29] lubricants were common choices. Various contact geometries were used, such as ball-on-disk [22], ball-on-flat [23], ring-on-disk, and pin-on-disk [24,25,26]. Substrates included low-carbon steels [22,23], high-carbon alloy steels [24,27], gray cast iron [25,26,28], and aluminum alloys [29]. Nickel coatings [22] or multilayer (CrN-Si:DLC multifunctional coatings) [23] were applied to low-carbon steels. Hard low-friction WC/C coatings or multilayer WC/C + DLC coatings [24] were applied to high-carbon alloy steels, and ceramic coatings were applied to gray cast iron and aluminum alloys, respectively [25,26,28,29].

Introducing new coating solutions to real compressors can enhance cooling capacity and improve volumetric efficiency while reducing energy consumption [30]. This aligns with global sustainability goals [31]. In oil-free nodes operating with gaseous refrigerants, WC/C coatings can significantly reduce the coefficient of friction by up to three times [24]. This reduction directly translates to lower energy consumption.

The primary objective of this study was to evaluate the potential application of WC/C coatings in the sliding friction pairs of refrigeration compressors. Through experimental testing, the study determined whether the WC/C coating could effectively function under specific operating conditions, particularly in environments with limited lubrication. The evaluation criterion was minimizing wear and friction resistance (coefficient of friction). Comparative tribological tests were performed using S235JR steel samples with and without the WC/C coating. The tests were conducted under conditions simulating extreme real-world scenarios for refrigeration compressors. These conditions included:

− dry operation—the compressor operated without oil in the friction nodes, relying solely on the refrigerant for lubrication,
− minimal lubrication—the compressor operated with minimal oil (only one drop) in the friction nodes, creating a starved lubrication environment.

## 2. Materials and Methods

### 2.1. Materials

**Figure 1:**
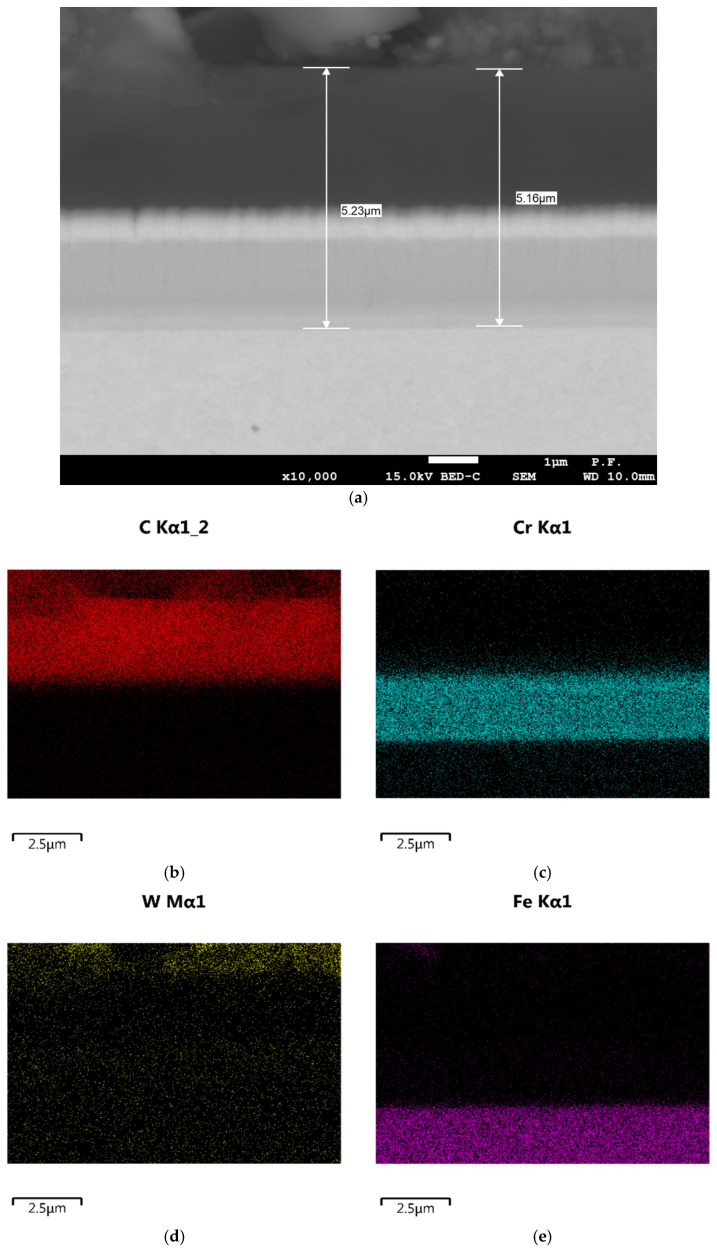
Transverse section of the WC/C coating: (**a**) thickness measurement; elemental distribution maps: (**b**) carbon, (**c**) chromium, (**d**) tungsten, (**e**) iron.

Figure 1 presents the model wear test node used. The samples made of S235JR steel were rectangular parallelepipeds with dimensions of 6 × 10 × 20 mm and an average surface hardness of 150 HV. Cylinders with a diameter of 20 mm and a width of 10 mm were used as counter samples. The counter samples were made of gray cast iron EN-GJL-250. Before each test, the samples were cleaned in an ultrasonic bath for about 15 min with an ultrasonic frequency of 45 kHz and ultrasonic power of 100 W.

WC/C coatings (amorphous hydrocarbons with tungsten doping) were obtained by plasma-assisted vapor deposition using magnetron sputtering at a temperature below 250 °C. Before applying the DLC coating, an intermediate chromium layer was deposited to improve the adhesion of the coating to the substrate. As a result of evaporation, atomized tungsten in the gas phase was deposited together with a gas containing a non-metallic carbon component, which was a coating component. Oerlikon Balzers produced the coatings. According to the manufacturer’s data, the WC/C coating has an instrumental hardness of 10–15 GPa. The thickness of the deposited WC/C layers was determined from a cross-section using a JSM 7100F scanning electron microscope (JEOL, Tokyo, Japan) equipped with an energy dispersive spectrometer (EDS) (Figure 1). A magnification of ×10,000 and an accelerating voltage of 15 kV were used. Microscopic observations of the cross-section reveal that the coating has a total thickness of approximately 5.2 µm and consists of three sublayers. Elemental distribution maps (Figure 1b–d) show that the approximately 3 µm thick near-surface layer consists mainly of carbon and tungsten atoms, with the highest concentration of tungsten atoms occurring very near the surface. The presence of carbon, chromium, and tungsten was observed in the transition layer (0.3 µm). The layer closest to the substrate, with a thickness of approximately 2 µm, is composed of chromium.

Friction and wear tests were conducted in the actual operating environment of the refrigeration compressor friction nodes with an oil-refrigerant mixture, or with the refrigerant alone. R452A refrigerant and a dedicated polyol ester (POE) oil with a viscosity grade (VG) of VG 32 were used in the tests. Table 1 shows the physical properties of the POE oils.

### 2.2. Method

**Figure 2:**
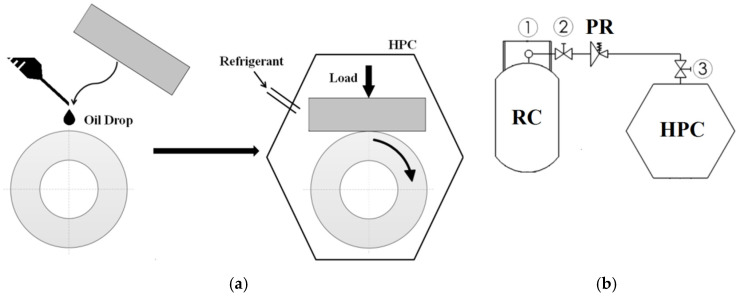
(**a**) Oil-refrigerant mixture preparation, (**b**) the instrumentation for supplying refrigerant: RC—refrigerant cylinder, PR—pressure reducer, HPC—high-pressure chamber, 1–3—ball valves.

The potential application of WC/C coatings in the sliding friction pairs of refrigeration compressors was evaluated using a tribometer equipped with a roller-on-block model node (Figure 2). The evaluation involved conducting comparative wear tests on coated and uncoated steel samples. The original research method for assessing the lubricating properties of oil-refrigerant mixtures in sliding friction nodes has been detailed in previous works [19,20]. Under the proposed concept, a comparative assessment of the tested materials was performed based on the volumetric wear of the sample in the roller-block configuration after friction tests in an oil-refrigerant mixture (Figure 2). Among the tested material variants, the one exhibiting lower wear demonstrated superior performance characteristics.

The procedure for testing the wear of sliding contact components in an oil-refrigerant mixture requires the creation of such a mixture in a test chamber equipped with a friction node. The oil-refrigerant mixture is formed through direct contact of both substances within a sealed chamber containing a model test node (Figure 2). The pressure in the test chamber for refrigerant R452A was 1.20 MPa, corresponding to the saturation pressure of refrigerant R452A at a temperature of 23 °C. An appropriate contact time between the two substances is required to obtain a saturated oil-refrigerant mixture. This time was determined in a series of preliminary tests. For the oil-refrigerant mixture (POE/R452A), the mixture formation time was set at 40 min [19].

The duration of the wear test was determined based on preliminary tests. The criterion adopted was to obtain a wear track with a width of at least 0.2 mm. Preliminary tests were conducted on samples with a WC/C coating under dry friction conditions. Based on a series of preliminary tests, the duration of the wear test was determined to be approximately 10 min.

Post wear tests, the wear tracks were observed using a JSM 7100F scanning electron microscope (JEOL, Tokyo, Japan) equipped with an EDS detector. Observations focused on the central region of each wear track.

The main test series are summarized in Table 2. Each series involved three wear tests on samples with (series 1–2) and without (series 3–4) the coating. The tests simulated harsh operating conditions in refrigeration compressors:

− dry operation (series 1 and 3): the friction nodes operated without any oil, solely in the refrigerant environment,
− minimal lubrication with an oil-refrigerant (POE/R452A) mixture (series 2 and 4): a small amount of oil (a single drop, approximately 30 mg) was added to the friction node in the chamber, creating a starved lubrication environment with the oil-refrigerant mixture.

All tests were conducted under a load of 60 N and a sliding speed of 0.5 m/s.

To estimate the volume of material removed from the block-shaped sample in the contact, the following relationship was used:

(1) $$V=0.5sr^{2}\left\{2\arcsin\left(\frac{x}{2r}\right)-\sin\left[2\arcsin\left(\frac{x}{2r}\right)\right]\right\}$$

where: *V*—volumetric wear of the sample [mm^3^], *s*—width of the sample (block) [mm], *r*—radius of the counter sample (roller) [mm], *x*—width of the wear track of the sample [mm].

During the tests, the contact torque was also measured. The coefficient of friction was determined from the value of the torque using the formula:

(2) $$\mu =\frac{M}{P\;r}$$

where *μ* is the coefficient of friction, [-], *M* is the torque [Nm], *P* represents the load (normal force), [N], and *r* is the radius of the roller, [m].

## 3. Results

**Figure 3:**
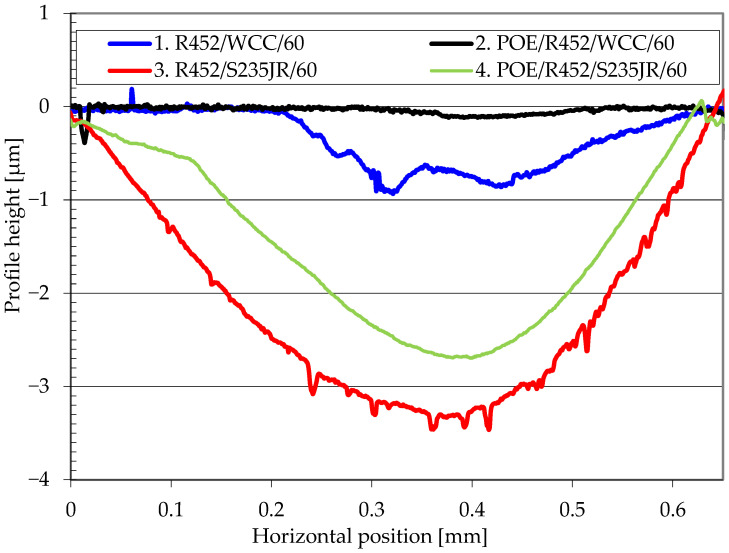
Wear track cross-section profiles.

**Figure 4:**
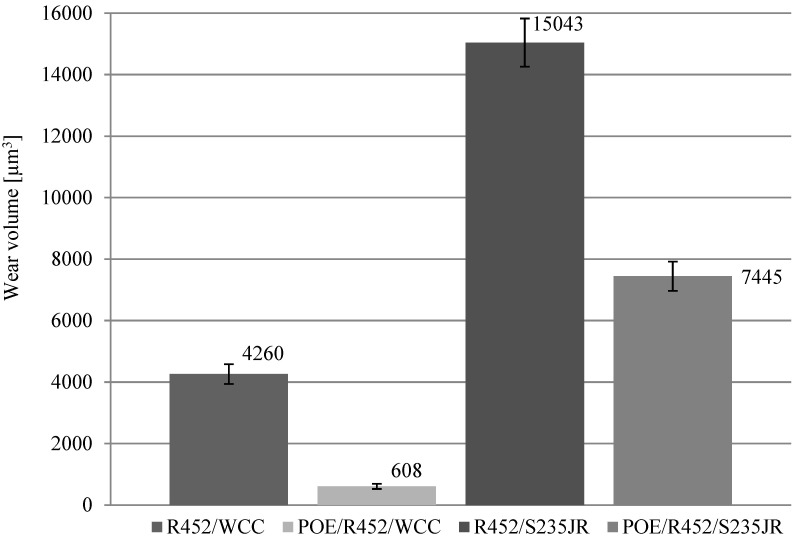
Wear volume results after tests.

**Figure 5:**
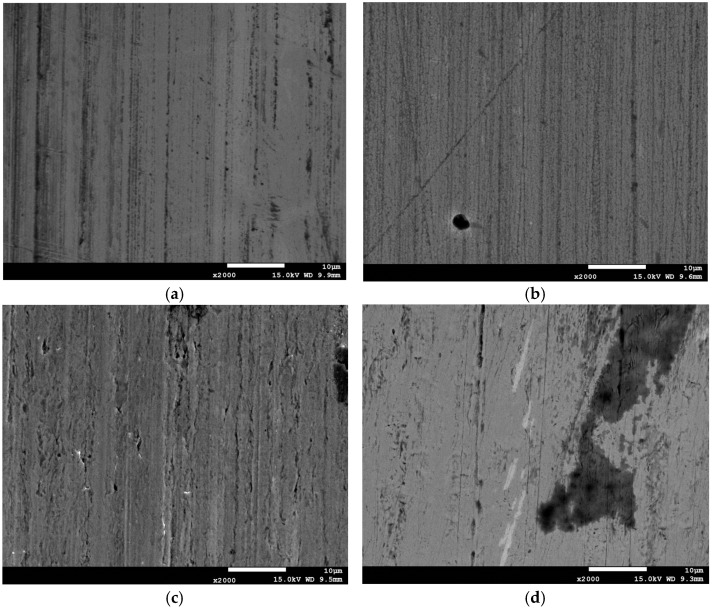
Surface wear marks after: (**a**) R452/WCC; (**b**) POE/R452/WCC; (**c**) R452/S235JR; (**d**) POE/R452/S235JR.

The potential of WC/C coatings to be used in the sliding contacts of refrigeration compressors was assessed based on the wear values and the friction coefficient observed during wear tests. Coated and uncoated samples were compared under extreme operating conditions. The results of the tests are presented in Figure 3, Figure 4 and Figure 5 and Table 3 and Table 4.

Figure 3 shows examples of the wear track profiles of the tested samples. The samples with the WC/C coating exhibited minimal wear (approximately 0.1 µm) under starved lubrication with the oil-refrigerant mixture (series 2). In contrast, samples with the WC/C coating tested without any lubricant (series 1) showed a track depth of about 0.9–1.0 µm. For steel samples without the WC/C coating, the wear track depth was 3.4 µm in the refrigerant environment (series 3) and approximately 2.6 µm under starved lubrication with the oil-refrigerant mixture (series 4).

Table 3 summarizes the wear test results, including wear track widths and depths and the estimated volumetric wear. The standard deviation for the volumetric wear was calculated based on three test repetitions. The obtained results are also illustrated in Figure 4.

For samples coated with WC/C, the volumetric wear was 4259 µm^3^ for series 1 (no oil) and 608 µm^3^ for series 2 (starved lubrication with an oil-refrigerant mixture). The volumetric wear for samples without the WC/C coating was 15,043 µm^3^ (series 3) and 7445.42 µm^3^ (series 4).

Table 4 shows the average friction coefficient values observed during the wear tests. For the combination of WC/C coated steel samples in the presence of the POE/R452A mixture, the coefficient of friction reached a minimum value of approximately 0.057. Under lubrication conditions with only the refrigerant, the coefficient of friction for the same samples was more than 65% higher.

Steel samples without coating had significantly higher coefficients of friction. They amount to 0.233 in starved lubrication with an oil-refrigerant mixture and approximately 0.251 in the presence of only the refrigerant. It is worth noting that for steel samples, the influence of the environment (presence of oil) on the coefficient of friction was significantly smaller (a difference of about 7%).

Observations of the wear track surfaces were conducted to identify the wear mechanisms of the sample’s base material and the WC/C coating under lubrication with only the refrigerant and under starved lubrication with an oil-refrigerant mixture. Example results are presented in Figure 5.

The wear tracks of the WC/C coated samples shown in Figure 5 indicate that the frictional forces did not cause the coating to delaminate from the substrate. No large pits or delaminations were observed. The wear track profiles presented in Figure 3 confirm that material loss occurred within the coating. The observed wear patterns are characteristic of abrasive wear. On the surface of the WC/C-coated samples, fine (although numerous) scratches from friction in the oil-refrigerant mixture and wider scratches from friction in the presence of only the refrigerant can be seen. In contrast, on uncoated S235JR steel samples, signs of furrowing were already visible, and after wear tests in an environment of only the refrigerant (R452A), individual pits appeared.

## 4. Discussion

The research results presented in this article indicate that WC/C coating significantly reduces sample wear—samples with the WC/C coating exhibited lower wear compared to uncoated steel samples. The difference amounts to approximately 72% in tests with the refrigerant R452 alone (comparison of series 3 (R452/S235JR) with series 1 (R452/WCC)) and about 92% under conditions of starved lubrication with the oil-refrigerant mixture (comparison of series 4 (POE/R452/S235JR) with series 2 (POE/R452/WCC)).

For samples with the WC/C coating, wear after tests using the oil-refrigerant mixture (series 2 (POE/R452/WCC)) was very low (difficult to quantify—Figure 2). It was significantly lower than in the other three series. It was 96% lower than the wear of the uncoated steel sample lubricated only with the refrigerant (series 3 (R452/S235JR)). The low volumetric wear may indicate the possibility of applying WC/C coating to the sliding friction nodes in refrigeration compressors. This low wear is probably the result of the high hardness of the coating and the synergistic interaction of the lubricant/lubricating environment with the coating.

Another positive effect of the interaction between the oil and the tested WC/C coating, from an operational standpoint, is the low coefficient of friction (Table 4). Even a small amount of oil (a single drop) allows for a reduction in sample wear. Most likely, this quantity is sufficient to obtain mixed friction conditions in the tested sliding node. This can be confirmed by the wear tracks of samples with the WC/C coating (Figure 5). Substantially narrower (albeit numerous) scratches were observed on the samples subjected to starved lubrication tests (series 2: POE/R452/WC/C) compared to those exposed to the refrigerant alone (series 1: R452/WC/C).

The WC/C coating provides very low wear and a low coefficient of friction under conditions of starved lubrication with the oil-refrigerant mixture. Diamond-like coatings in tribological contact can undergo graphitization in the contact area [32]. The presence of the graphite phase reduces friction, as graphite is a solid lubricant. Most likely, this mechanism of lowering the friction coefficient also occurs under the operating conditions of refrigeration compressors (lubrication with an oil-refrigerant mixture under starved lubrication conditions). Graphitization can be induced by suitable thermo-mechanical conditions, i.e., the temperature increase in the frictional contact zone and high shear stresses under starved lubrication conditions.

## 5. Conclusions

The primary practical outcome of this research is the experimental validation of WC/C coatings for sliding friction pairs in refrigeration compressors. The sliding pairs operate in a challenging environment characterized by starved lubrication with an oil-refrigerant mixture, often leading to high temperatures and significant mechanical stress. According to previous studies [5,10], such conditions can hinder the performance of WC/C coatings; therefore, this study aimed to experimentally confirm the effectiveness of these coatings in mitigating wear and reducing friction coefficients under extreme operating conditions.

The study findings demonstrate that WC/C coatings are suitable for use in the sliding components of refrigeration compressors, even in conditions where lubrication is sparse. Importantly, these wear tests were conducted using the eco-friendly refrigerant R452A. Several factors support the use of WC/C coatings in refrigeration compressors, including the following:

1. Minimal wear—samples with the WC/C coating exhibited the least wear under starved lubrication conditions with the oil-refrigerant mixture and after friction in the presence of the refrigerant alone. The tested coating can provide good protection for the sliding friction nodes in refrigeration compressors in extreme operating conditions resulting from a lack of oil.
2. Low coefficient of friction—in a sliding pair with a sample coated with WC/C under starved lubrication with the POE/R452 mixture, the lowest coefficient of friction was observed (approximately 0.06). This value indicates that even a small amount of oil (one drop) is most likely sufficient to obtain mixed friction conditions in the tested sliding node and reduce material loss. Such a course of frictional interactions is confirmed by tracks of mild abrasive wear (shallow scratches).

## Figures and Tables

**Table 1:** Properties of investigated polyol ester (POE) oils.

Oil Type	Property				
	Kinematic Viscosity (mm^2^ s^−1^)		Density at 15 °C (kg m^−3^)	Flash Point (°C)	Pour Point (°C)
	40 °C	100 °C			
POE1	32	6.0	981	250	−54

**Table 2:** Summary of research series.

Series Number	Lubricant	Material	Load [N]	Indication
1	R452A	S235JR + WC/C	60	R452/WC/C
2	POE/R452A	S235JR + WC/C	60	POE/R452/WC/C
3	R452A	S235JR	60	R452/S235JR
4	POE/R452A	S235JR	60	POE/R452/S235JR

**Table 3:** Wear test results.

Series Number	Series Abbreviation	Wear Track Width [µm]	Wear Track Depth [µm]	Wear Volume [µm^3^]
1	R452/WC/C	440	1.0	4259 ± 321
2	POE/R452/WC/C	230	0.1	608 ± 83
3	R452/S235JR	670	3.4	15,043 ± 784
4	POE/R452/S235JR	530	2.6	7445 ± 476

**Table 4:** Average values of friction coefficients.

Series Number	Series Abbreviation	Average COF
1	R452/WCC	0.164 ± 0.029
2	POE/R452/WCC	0.057 ± 0.027
3	R452/S235JR	0.251 ± 0.010
4	POE/R452/S235JR	0.233 ± 0.005

## Data Availability

The original contributions presented in the study are included in the article, further inquiries can be directed to the corresponding author.

## List of Symbols and Abbreviations

AlCrN	Aluminum Chromium Nitride
CVD	chemical vapor deposition
COF	coefficient of friction
CrN	chromium nitride
DLC	diamond-like carbon
EDS	energy-dispersive
GWP	global warming potential
MoS_2_	Molybdenum disulfide
PACVD	plasma-assisted chemical vapor deposition
PAG	polyalkylene glycol-based lubricants
POE	polyol ester-based lubricants
PVD	physical vapor deposition
R125; R134a; R32; R404A; R410A; R452A; R600a; R744; R1234yf	refrigerants
TiN	titanium nitride
TiAlN	Titanium aluminum nitride
VG	viscosity grade
WC	tungsten carbide
WC/C	diamond-like carbon coating doped with tungsten, a-C:H:W
